# The Views of Swim School Providers on Impacts of a Population‐Level Swimming Lesson Voucher Program

**DOI:** 10.1002/hpja.70177

**Published:** 2026-03-21

**Authors:** Amy E. Peden, Edwina Mead, Rona Macniven

**Affiliations:** ^1^ School of Population Health UNSW Sydney Kensington New South Wales Australia; ^2^ UNSW Beach Safety Research Group UNSW Sydney Kensington New South Wales Australia

**Keywords:** aquatic activity, drowning prevention, physical activity, policy

## Abstract

**Issue Addressed:**

Learning to swim reduces drowning risk and facilitates access to lifetime health benefits of aquatic activity. Amid cost‐of‐living pressures and disparities in swimming lesson access, further exacerbated by the COVID‐19 pandemic, governments have invested in strategies to foster access and participation. This study examines the swim school industry's perceived impacts of the NSW government First Lap voucher program to subsidise swimming lessons for young children.

**Methods:**

A mixed‐methods approach analysed anonymous survey responses of 100 swim schools. Quantitative analysis calculated frequencies with 95% confidence intervals (95% CI), and thematic analysis was applied to open‐ended qualitative responses.

**Results:**

Most providers (63%) reported increased enrolment (95% CI 53%–72%) and 58% noted boosted income (95% CI 48%–67%) since program commencement. Sixty‐nine percent observed more new enrolments (95% CI 60%–78%), leading to additional classes (57%; 95% CI 47%–67%) and teaching staff hours (59%; 95% CI 49%–68%). Increased pool space usage (36%; 95% CI 27%–46%) and non‐teaching staff hours (36%; 95% CI 26%–45%) were noted. Key themes derived from open‐ended question responses included swim schools' operational impacts (such as promotional opportunities, student retention and increased administration) and family effects, such as improved affordability and earlier enrolment.

**Conclusions:**

Findings highlight benefits such as increased enrolment and income, alongside industry challenges in staffing and resourcing. Future research should explore voucher programs' impact on participant retention and long‐term enrolment outcomes.

**So What?:**

This study details impacts of a population‐level voucher program on the aquatic industry, addressing a knowledge gap around the role and views of industry, which can inform future health promotion strategies.

## 1 Introduction

Swimming and other aquatic activities are known to have significant benefits for physical and mental health and well‐being [1, 2]. Beyond these benefits, learning to swim is also a key strategy to reduce drowning risk [3]. Drowning is a leading killer of young children in all regions of the world [4, 5], and the acquisition of basic swimming and water safety skills in childhood can confer protection, contributing to a lifetime of safe and enjoyable aquatic activity [6].

The provision of swimming lessons is a key facet of operations at many public pools and aquatic facilities [7]. This important education contributes to the health, social and economic value of the aquatic industry [8]. This includes contributing to the reduced burden of disease in Australia by promoting physical activity and averted social and economic losses through reduction of child drowning through swimming education [9].

However, as with many other industries, the COVID‐19 pandemic caused significant disruption to the aquatic industry. In Australia, as in many other countries, COVID‐19 related lockdowns manifested in temporary pool closures, missed lessons, swim teacher shortages and long wait lists for lessons when pools re‐opened [7]. Given research shows low skill attainment against national benchmarks [10] and withdrawal from lessons prior to achieving minimum benchmarks [7], the industry strongly advocated for initiatives to encourage swim lesson participation [11].

The COVID‐19 pandemic also further exacerbated known determinants of health impacting participation in swimming lessons. Research focused on school‐aged children has shown children from low socio‐economic areas and regional areas were underrepresented in analysis of private learn‐to‐swim data [12]. Similarly, parents report 10% of children aged 5–14 years have never attended swimming lessons, with these children more likely to be from low socio‐economic and regional and remote areas [13]. To address concerns about disparities of access and disruption to children's swimming lessons, the New South Wales (NSW) Government launched the First Lap voucher program in December 2021. The program provided two AU$100 vouchers, one per financial year in 2021–2022 and 2022–2023, for parents and carers of children aged between 3 and 6 years of age [14]. The program aimed to increase participation in learn‐to‐swim programs for this age group, while also raising awareness among parents and caregivers of this age group about the importance of early swimming education [15].

To access the program, parents or caregivers of children within the target age range were required to register for a voucher via the NSW Government's Service NSW portal. Vouchers could then be redeemed for a minimum of five lessons with a registered provider for either intensive programs or weekly/term‐based lessons [15]. The voucher could be used to facilitate a new enrolment or to offset costs of ongoing enrolment. After the initial 2 years, the program shifted to a lower value $50 voucher which expired on the 30th June 2024.

A comprehensive evaluation of the program [15] including parents and caregiver surveys and government captured voucher registration and redemption data was conducted [16]. As a component of the evaluation, this study examines the responses to a survey of swim school providers to capture the perceived provider‐side impacts of the First Lap voucher program.

## 2 Methods

### 2.1 Survey Design and Recruitment

All swim school providers who had been onboarded to the First Lap program by December 2022 as registered providers were invited to participate in a short online survey about their experiences. An invitation to complete the survey was issued via email by the NSW Government to one contact email address per registered provider. Instructions advised that only one staff member per provider complete the survey. The survey was administered via an NSW government survey portal.

The survey was developed in line with the evaluation logic model [15] and using expert opinion of the evaluation team, including authors A.E.P. and R.M., as well as insights from NSW Government. The survey included questions about what proportion of children aged 3–6 years redeemed a voucher, and changes in enrolment levels, number of classes, pool space used and staff employment. See Box 1 for the provider survey questions. The online survey was pilot tested among the evaluation team for comprehension and functionality and refined prior to data collection commencing.

BOX 1 **Provider survey questions.**

- What is your role at the organisation? (multi‐select: facility manager; general manager; business owner/operator; swim school manager; administration/finance; swim teacher; other please specify)
- Approximately what proportion (%) of children aged 3–6 years enrolled in learn to swim lessons at your swim school have redeemed a voucher since the First Lap program began in December 2021? (choose one: Less than 10%; 10%–24%; 25%–49%; more than 50%)
- Has the First Lap voucher scheme increased enrolment in learn to swim lessons for children 3–6 years at your swim school? (choose one; yes; no; unsure)
- Has the First Lap voucher program resulted in any of the following changes in learn to swim lesson operation at your venue?
  ○ More classes taking place (yes; if yes approximately how many more; no)
  ○ More pool space being used (yes; if yes what % more; no)
  ○ Increased child enrolment (yes; if yes approximately how many new enrolments; no)
  ○ Increased number of teachers employed (yes; if yes how many new teachers; no)
  ○ Increased hours for existing staff (swim teachers) (yes; if yes, approximately how many hours per week; no)
  ○ Increased hours for existing staff (non‐swim teachers) (yes; if yes, approximately how many hours per week; no)
  ○ Increased swim school income (yes; if yes, approximately by how much % increase; no)
- Has the First Lap voucher program resulted in any other changes in learn to swim lesson operation at your venue? (free text response)

### 2.2 Data Analysis

The study utilised a mixed methods approach to analysis comprising both quantitative and qualitative methods. Data were downloaded as an Excel file from the NSW government survey portal (SurveyManager) after the survey had closed by a member of the research team.

Closed questions with yes/no answers were analysed using a binomial proportion to calculate a 95% confidence interval (CI) using Python with StatsModels 0.14.4 [17, 18]. Fisher's exact test was used to test for significance when comparing groups.

Inductive thematic analysis [19] and sentiment analysis were performed on the final question, an open‐ended question which invited the provider to comment on “any other changes in operation” caused by the First Lap program. Data were visually grouped using mind mapping [20] and then thematically coded in Excel. Thematic analysis was conducted by a member of the research team and independently validated by the other authors. Quotes are provided verbatim to enhance understanding of identified themes, presented alongside respondent role.

### 2.3 Ethics

The broader evaluation of the First Lap voucher program, and the current study, obtained university ethics approval (ID: HC220282). All registered providers were contacted by email to invite them to participate in the provider survey and were asked to click a link to access the Participant Information Sheet and Consent Form for more information and to give consent to take part by starting the survey.

## 3 Results

### 3.1 Quantitative Results

Of the 518 providers who had been onboarded at the time of survey administration, 100 (19.3%) completed the survey. Responses were received from Business owner/operators (*N* = 72; 73.5%), Swim school managers (*N* = 27; 27.6%), Swim teachers (*N* = 23; 23.5%), Facility managers (*N* = 13; 13.3%), Administration/finance (*N* = 15; 15.3%), General manager (*N* = 2; 2.0%), President of swim club *(N* = 1; 1.0%), and Program leader (*N* = 1; 1.0%) (Table 1). Twenty‐five percent reported multiple roles, commonly business owner operator and swim teacher (*n* = 17; 68.0% of those with multiple roles).

**TABLE 1:** Swim school provider quantitative survey responses.

Question	Response	Number of responses
What is your role at the organisation?	Facility manager	13
	General manager	2
	Business owner/operator	72
	Swim school manager	27
	Administration/finance	15
	Swim teacher	23
	Other	2
Approximately what proportion (%) of children aged 3–6 years enrolled in learn to swim lessons at your swim school have redeemed a voucher since the First Lap program began in December 2021?	< 10%	5
	10%–24%	18
	25%–49%	27
	> 50%	49
	No response	1
Has the First Lap voucher scheme increased enrolment in learn to swim lessons for children 3–6 years at your swim school? (1)	Yes	62
	No	19
	Unsure	18
	No response	1
Has the First Lap voucher program resulted in any of the following changes in learn to swim lesson operation at your venue?		
More classes taking place	Yes	57
	No	43
	No response	0
More pool space being used	Yes	36
	No	63
	No response	1
Increased child enrolment	Yes	68
	No	31
	No response	1
Increased number of teachers employed	Yes	39
	No	58
	No response	3
Increased hours for existing staff (swim teachers)	Yes	58
	No	41
	No response	1
Increased hours for existing staff (non‐swim teachers)	Yes	35
	No	64
	No response	2
Increased swim school income	Yes	57
	No	42
	No response	1

**FIGURE 1:**
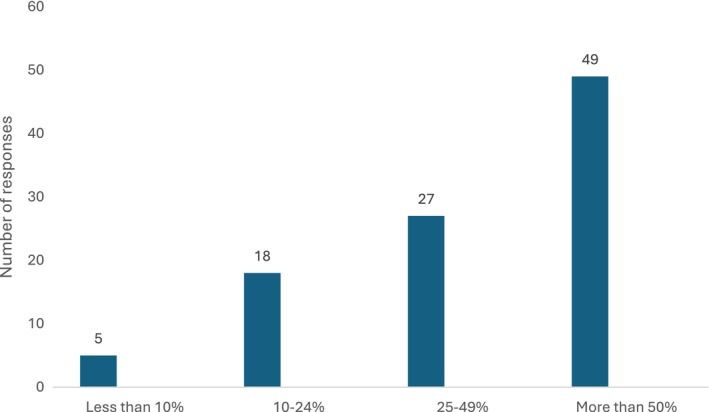
Proportion of children aged 3–6 years enrolled at swim school who redeemed a First Lap voucher (*n* = 99).

When asked what proportion of children aged 3–6 years enrolled in learn‐to‐swim lessons at their swim school had redeemed a voucher since the First Lap program began in December 2021, 49 respondents (49.5%) reported more than 50% of enrolees. Just five respondents (5.1%) reported less than 10% of enrolees redeemed a voucher (Figure 1).

Most providers (63%) indicated that the voucher program had increased enrolment at their facility (95% CI 53%–72%) and had resulted in increased income (58%; 95% CI 48%–67%). Most also said that there had been an increase in new enrolments (69%; 95% CI 60%–78%), and that more classes were taking place (57%; 95% CI 47%–67%), which meant that hours were increased for swim teaching staff (59%; 95% CI 49%–68%). A minority expressed that more pool space was being used (36%; 95% CI 27%–46%), and that non‐teaching staff hours had increased (36%; 95% CI 26%–45%) (Table 2).

**TABLE 2:** Swim school provider affirmative responses to the question “Has the First Lap voucher program resulted in any of the following changes in learn to swim lesson operation at your venue?”

Sub‐question (number of responses)	Proportion answering affirmatively (95% confidence interval)
Increased enrolment in learn to swim for children aged 3–6 (99)	0.63 (0.53–0.72)
More classes taking place (100)	0.57 (0.47–0.67)
More pool space being used (99)	0.36 (0.27–0.46)
Increased child enrolment (99)	0.69 (0.60–0.78)
Increased number of teachers employed (97)	0.40 (0.30–0.50)
Increased hours for existing staff (swim teachers) (99)	0.59 (0.49–0.68)
Increased hours for existing staff (non‐swim teachers) (99)	0.36 (0.26–0.45)
Increased swim school income (98)	0.58 (0.48–0.67)

### 3.2 Qualitative Results

Eighty providers recorded a response to the final open‐ended question “Has the First Lap voucher resulted in any other changes in learn to swim at your venue?” however, 12 responses were discarded as they contained only the word “no.” Compared to non‐responders, the remaining 68 providers with responses to this question more often reported that “More than 50%” of eligible children at their services were using First Lap vouchers (59% vs. 33%; *p* = 0.02), but less often reported that the program had increased enrolment (53% vs. 82%; *p* = 0.005). No significant differences were found by provider type. Most responses were 1–2 sentences long, with a minority (14; 21%) having three or more. Due to the brevity of responses, most responses (41; 60%) were categorised with a single theme. Two major themes were found among the comments entered. These were effects on the swim school operations and observed effects on families.

#### 3.2.1 Effects on Swim School Operations

Comments made regarding perceived impact on swim school operations spanned four sub‐themes including positive impacts (opportunities to promote the swim school), mixed (student retention and demand), neutral (voucher was mostly used by current customers and no effect) and negative (increased administration). The majority of these comments (*n* = 38) reported the program had a positive effect on swim school operations, while 22 indicated a neutral effect, and 13 a negative effect (some comments indicated a combination of effects). Each of the sub‐themes is now presented.

##### 3.2.1.1 Opportunity to Promote the Swim School

The First Lap program helped promote some swim schools as it “Increased the community profile of the facility” (Facility manager) while for others being listed as a registered provider enhanced visibility of the swim school within the community as families searched to find a provider they could use the voucher with: “Increased advertising of my business as families are looking for First Lap Voucher providers” (Multiple roles).

Others reported using the First Lap voucher in their own promotions “We could advertise the program with [the First Lap voucher] as an incentive” (Facility manager) with such actions increasing parent/caregiver engagement with learn to swim providers: “We are now getting loads of enquiries and questions—particularly from parents of Covid babies, who show timidness toward the water and new challenges. It's great for encouraging them to learn this important life saving skill” (Business owner/operator).

##### 3.2.1.2 Mixed Impact on Demand

Twenty‐four providers who answered the question indicated increased demand as a result of the voucher program. One provider stated “I've taken on more younger kids to accommodate the use of the voucher” (Business owner/operator) while another reported the voucher had resulted in “More beginner classes” (Multiple roles). Some who were already enrolled in lessons used the voucher for additional classes indicating “Current enrolments booking extra lessons than they usually would” (Business owner/operator).

In addition, two providers indicated that demand was increased in other aspects of their facility's operation. Two indicated that squad training (advanced or competitive skill building) enrolment had also increased with one noting “Higher participation in swimming club and squad training” (Swim teacher).

Three providers commented that overall entries to their facility had increased, reporting “more swimming entries to our facility” (Swim school manager) and for one, the increased demand resulting from the voucher program reversed previous reductions in visitation: “It has increased visitation that had been declining” (Other—program leader).

However, the increase in demand was such that nine swim schools reported being overwhelmed and unable to meet it: “We operate at full capacity with a waiting list” (Multiple roles). There were two major reasons given for this, with the first being facility access/capacity constraints based on available hours of operation:

“We often have to direct people to other providers as we are at capacity and already at maximum hours open.” (Multiple roles)

“We are unfortunately limited to 5 hours of pool access per week and we are at full capacity with no opportunity to expand.” (Business owner/operator)

The second reason for being unable to meet the demand generated by the voucher program related to not enough teaching staff with one provider reflecting the program “may have had a bigger impact if I was able to get more staff to teach” (Multiple roles). Another reflected on the scheme being introduced amid a sector already struggling with workforce challenges, stating that “We simply don't have the instructors to be offering additional (or in some cases, normal) classes” (Multiple roles).

Providers indicated challenges around staffing post COVID‐19 pandemic‐related lockdowns have put the industry further under pressure to respond to increased demand associated with the vouchers:

“Staff levels are a major issue across the industry without them we can't provide classes for those wishing to use their vouchers.” (Multiple roles)

“These vouchers have caused more demand than we can accommodate, especially after COVID shutdowns and losing staff in the industry.” (Multiple roles)

Two providers went further and suggested that additional funding should be directed to teacher training:

“Funding for swim schools to employ more staff would assist in the increase of children's swimming lessons across the board.” (Multiple roles)

“Would also be great if more money was invested in supporting the promotion, training and hiring of Instructors through RLS [Royal Life Saving] and AustSwim [providers of teacher training in New South Wales].” (Multiple roles)

##### 3.2.1.3 Mixed Effect on Retention

In addition to affecting demand, nine providers also commented that the voucher program had aided learner retention: “Parents have renewed and stayed in the program longer due to First lap program” (Swim school manager) Providers also reported the voucher program encouraged learners to commence swimming lessons earlier, leading to longer‐term retention:

“Once the child is enrolled they usually stay in their lessons until they are competent swimmers which results in students staying in the program for longer as they are starting earlier.” (Multiple roles)

Specifically, the voucher program appears to have offset barriers to retention around cost. This has also had the dual benefit of children attending their learn to swim lessons more consistently:

“Parents were hardly turning up to lessons and not enrolling their child because they couldn't afford it. Then they used First Lap vouchers and came regularly. They saw the improvement in their child's swimming ability and continued their enrolment after the voucher had run out.” (Business owner/operator)

“It has made sure family from less affluent background have put their children more consistently in the classes and not taken them in and out because of budget restraints.” (Business owner/operator)

On the other hand, three providers reported that the voucher program seemed to decrease learner retention with children being unenrolled when the voucher ran out:

“Unfortunately a good percentage of people only enrolled for the freebie.” (Facility manager)

This caused frustration around class planning:

“I feel it has reduced the retention rate leaving large gaps in classes. Some, not all, but about 15% of new enrolments tend to use first lap vouchers and then withdraw once voucher has been used.” (Business owner/operator)

Another expressed concern at the short duration of swimming education children in these scenarios received at their swim school:

“Parents were enrolling children only to use first lap voucher which for our centre was only a month worth of lessons. After that customers never came back. More regulations on how they have to be used [are needed] as four lessons are not enough to teach children to swim.” (Business owner/operator)

##### 3.2.1.4 Voucher Was Mostly Used by Current Customers

Seven providers noticed that the primary users of the voucher were their current customers: “Most of our current customers used the First Lap program” (Business owner/operator), rather than introducing new customers to the benefits of learning to swim: “The children who used them would be attending anyway” (Business owner/operator).

This was perceived to be due to a variety of reasons, including lack of capacity to take on new customers due to workforce shortages:

“People are just substituting payment with a voucher. It still hasn't fixed the lack of Learn to Swim Instructors that are in short supply Australia wide.” (Business owner/operator)

Another reason was perceived to be due to a lack of awareness of the First Lap program in the community:

“We had to make sure people learned about the amazing opportunity to use the vouchers towards lessons as almost nobody knew about the program.” (Business owner/operator)

Increased promotion was suggested as a strategy to increase awareness of both the voucher program, but also swimming lessons in general:

“We would love to see the NSW government promoting First Lap vouchers and the importance of swimming lessons more.” (Business owner/operator)

##### 3.2.1.5 No Effect

Six providers noted that the voucher had little effect on their operations reporting “our classes are the same, and our child to [teacher] ratio has not changed either” (Business owner/operator) or believing any changes were not due to the voucher program:

“We have increased our numbers because a teacher returned to the pool. People come to my pool because of the reputation rather than being able to use a voucher” (Multiple roles)

##### 3.2.1.6 Increased Administration

Ten providers chose to make comments that the administration work was significantly increased for redeeming First Lap vouchers saying it's “just more administration for staff to process and redeem” (Facility manager) with two providers reflecting that the process was income negative due to the additional administrative burden believing the program generated “more paperwork that outweighs extra income” (Business owner/operator) because “it has resulted in more admin hours for our team” (Facility manager).

Five also noted that differences in the eligibility, conditions and redemption process between First Lap and Active Kids vouchers (another program run by the NSW government to increase physical activity in primary school aged children) increased administration complexity:

“With the First Lap and the Active Kids both needing to be submitted differently and using different websites/apps this just increases admin time and is frustrating.” (Business owner/operator)

“It would be great if the ‘First Lap’ could be ALL swimming levels ‐ would simplify the process for Swim Schools a lot if we didn’t have to juggle both Active Kids and First Lap Vouchers.” (Multiple roles)

One provider reflected that the administrative burden associated with First Lap was lower than Active Kids but still posed a challenge “The First lap voucher process has been easier on administration than the Active kids vouchers but it still requires work arounds in systems which increases admin staff workload who are already overworked due to shortages” (Multiple roles). Feedback on future improvements focused on user experience to streamline both NSW government voucher schemes: “Would be much better if these departments [First Lap and Active Kids] could work together to create a more user friendly and more streamlined approach” (Business owner/operator).

#### 3.2.2 Observed Effects on Families

Twenty‐seven providers also made comments on how their customers were positively affected by the program, with two sub‐themes around affordability for families and increased awareness resulting in enrolment at a younger age.

##### 3.2.2.1 More Families Can Afford Lessons

One of the mechanisms of how First Lap increased enrolments was through increasing the number of families who could afford to have their child attend swimming lessons: “More kids being able to continue to swim who couldn't afford to do lessons” (Swim teacher) including low‐income earners: “This just makes it a little easier for those on low incomes” (Business owner/operator) and groups that may not traditionally access lessons: “So many vulnerable families who couldn't otherwise afford or access learn to swim have been utilising the program. It's been amazing to be able to offer classes to a more diverse range of people” (Business owner/operator).

Another provider indicated the voucher helped families enrol multiple children in swimming lessons, which previously may have been cost‐prohibitive:

“More families being able to afford more than one child swimming.” (Multiple roles)

The vouchers also supported families in being able to continue to attend swimming lessons, when cost may have been a factor in ceasing this:

“Increase in those who normally could not afford swimming lessons to participate and/or stay in lessons longer.” (Business owner/operator)

“With financial hardships parents continue with their booking because the voucher pays a part.” (Facility manager)

“What it has done is take some of the financial burden of fees off the parents which is a wonderful thing.” (Swim school manager)

Twenty providers also commented that parents appreciated the financial assistance with one provider saying “patrons very appreciative of reduced impact on their personal finances” (Multiple roles), while another said “parents are really appreciative and supportive of the programme” (Business owner/operator).

The voucher was also attributed with introducing new families to learn‐to‐swim opportunities addressing affordability as a barrier: “It has exposed the swim school to new families who may not have attended lessons because they couldn't afford it” (Business owner/operator). Another provider reflected that addressing financial barriers to children attending their first class could lead to continued engagement in swimming lessons: “The biggest thing that First Lap has done is broken down the barrier holding parents from getting into their first class. Once they try for the first time and their child enjoys it, they stay” (Multiple roles).

##### 3.2.2.2 Increased Awareness Such That Families Enrolled Their Children at Younger Ages

Seven providers noted that the First Lap program resulted in “More awareness of swimming lessons for younger children” (Multiple roles). This was also evident in the voucher program encouraging enrolment of other children in the family: “We are getting the younger siblings enrolled” (Multiple roles).

This resulted in some families enrolling their children at younger ages, perceived to create improved swimming skills: “Parents are booking children in at an earlier age and therefore the children's swimming skills are improving at an earlier age … most children who start swimming at 3 years old or younger can swim independently in the water without floaties before they go to school” (Swim school manager).

## 4 Discussion

Part of the broader program evaluation [15], this study explored the views of providers on impacts of a voucher program to subsidise swimming lessons for pre‐school aged children in the Australian state of NSW. Amidst various policies aimed at addressing the challenges around encouraging enrolment in learn to swim, as well as broader participation in swimming, examining the impact on providers addresses an important gap in the literature to date [21, 22, 23].

Findings indicate providers reported both positive, negative and mixed impacts of the program. Most of the respondents indicated that the program had increased enrolments and 58% reported increased income. Sixty‐nine percent indicated increases in new enrolments, an aim of the program [15]. However, while many of the comments were positive, qualitative responses identified concern from some providers about the ability to adequately support increasing demand, including the administrative burden of voucher redemption and reporting. The voucher program was implemented shortly after a period of COVID‐19‐related lockdowns in Australia which exacerbated industry challenges around teacher shortages [24], though a policy supporting free swim instructor training was launched in response to demand generated by the voucher program [25]. While the increased income reported by 58% of providers will assist in strengthening the industry, sustainable strategies to support recruitment and training of teaching staff into the future need to be examined, particularly in light of the challenges faced in regional and remote areas and in towns with seasonal facilities [7]. Similar findings from sport and recreation voucher program evaluations provide further support for the need to consider sector capacity, including early preparation and administrative challenges, ahead of implementation [26].

Cost has been identified as a barrier to participation in learn to swim lessons in Australia [27, 28], so it is pleasing the main impact providers reported within the “effects on families” theme was “more families able to afford lessons/fee relief.” However, this was countered by provider perspectives that the voucher was predominately being used by those already enrolled in learn to swim. This is a finding mirrored in evaluation research conducted with voucher recipients regarding voucher redemption [16] and was also seen in the UK's free swimming program [23]. This further highlights the social determinants of health‐based inequities in swimming lessons access, with previous research finding children from high income and metropolitan locations more likely to participate in private swimming lessons [12]. It has been recommended that future programs are means‐tested, providing more support to those who need it most, rather than a universal approach. A recent policy directs funding for subsidised swimming lessons to communities at increased risk of drowning [29]. The impacts of any new policies should be fully evaluated and engage with providers in determining evaluation metrics [30].

Although the program met its aim of increased enrolments, responses from some providers indicated concern about decreasing retention, reporting some patrons ceased participation after the voucher‐subsidised lessons. Further research is needed to determine if early exposure to swimming lessons encourages participation at an older age. This will be important given the minimum of five free lessons provided by the voucher, which would result in little actual skill attainment, conferring no protection from drowning for those in a high‐risk age group [4, 31]. Further, for those children who only received the minimum of five lessons and did not stay enrolled in swimming lessons post their voucher being redeemed, there may be an increased risk of drowning conferred by increased confidence around water among young children as well as decreased attentive supervision among caregivers, although there is no current evidence supporting these concerns. On the contrary, parental surveys undertaken as part of the First Lap program evaluation highlighted knowledge gain regarding child drowning prevention strategies, including statistically significant improvements in awareness of supervision as a strategy [32].

Additionally, this finding comes amidst broader concerns about the swimming skills of Australian children. Recent research [13] has found a significant drop off in swimming lesson participation after 7 years of age, and 46% of Year 6 children (nominally 11–12 years of age) do not meet the minimum skills for their age as documented in the National Swimming and Water Safety Framework [10]. Longer term enrolment trends in and around temporary policies such as First Lap should continue to be monitored. Ongoing or alternative strategies are also required to encourage enrolment among older children who have never participated in lessons, or who have not yet achieved minimum standards [10] to ensure all children are supported to attain these skills prior to adulthood.

## 5 Strengths and Limitations

This study examines impacts of government policy on industry, a group rarely examined in research regarding such initiatives [22, 23]. Further, insights derived via both quantitative and qualitative data can support development of future programs. However, this research should be considered in the context of some limitations. Although the survey was kept deliberately short to encourage completion, only a fifth of registered providers completed the survey. This meant that the survey did not collect information about facility characteristics, which limits understanding of how representative the results are of the broader group of registered providers, nor the broader aquatic industry in New South Wales. The survey was conducted at the end of the first year of a 2‐year program, and increased registration and redemption of vouchers in the second year of the program [16] is likely to have led to bigger impacts on providers, not captured in these results. As the program ceased in June 2024, it is not feasible to conduct further surveys, although future research could consider the utility of exploring any residual effects of the voucher program on the industry. The survey was anonymous and not able to be linked to the list of registered providers, limiting ability to link to other provider information. Further, due to the anonymous nature of the survey, we are unable to determine if duplicate responses from the same provider were received, though the survey did not capture any duplicate IDs.

## 6 Conclusion

This research has identified both positive and negative impacts on swim school providers regarding the implementation of a voucher program to subsidise swimming lessons for pre‐school aged children in the Australian state of NSW. Findings can inform the creation of subsequent policies and large‐scale programs aimed at improving swimming lesson participation for children, as well as better enabling providers to support these initiatives.

## Funding

This work was supported by the New South Wales Government under grant RG214380. A.E.P. is funded by a National Health and Medical Research Council (NHMRC) Emerging Leadership Fellowship (Grant ID: APP2009306).

## Ethics Statement

The broader evaluation of the First Lap voucher program, and the current study, obtained ethics approval from the University of New South Wales Human Research Ethics Committee (ID: HC220282).

## Conflicts of Interest

The authors declare no conflicts of interest.

## Data Availability

The data that support the findings of this study are available from the corresponding author upon reasonable request.

## References

[1] S. McNally , “Boosting Swimming for Health and Joy,” British Medical Journal 384 (2024): q393. 38378195 10.1136/bmj.q393

[2] K. Overbury , B. Conroy , and E. Marks , “Swimming in Nature: A Scoping Review of the Mental Health and Wellbeing Benefits of Open Water Swimming,” Journal of Environmental Psychology 90 (2023): 102073.

[3] World Health Organization , Preventing Drowning: An Implementation Guide (World Health Organization, 2017).

[4] A. E. Peden , R. C. Franklin , and T. Clemens , “Can Child Drowning Be Eradicated? A Compelling Case for Continued Investment in Prevention,” Acta Paediatrica 110, no. 7 (2021): 2126–2133. 33043488 10.1111/apa.15618

[5] World Health Organization , Global Status Report on Drowning Prevention (World Health Organization, 2024).

[6] M. C. Watson and K. E. Neil , “Promoting Swimming: A Positive Approach to Public Health,” BMJ 384 (2024): q393. 38471719 10.1136/bmj.q606

[7] PwC Australia , “Towards a Water‐Loving Nation Free From Drowning: The Role of Learn to Swim,” 2022, Prepared for Royal Life Saving, Australia.

[8] C. Yeomans , R. Storr , E. Sherry , and A. Karg , “Social Value Accumulation Through Australian Aquatic Facilities,” Managing Sport and Leisure (2024): 1–16.

[9] PwC Australia , “The Health, Social and Economic Value of the Australian National Aquatic Industry,” 2021, Prepared for Royal Life Saving, Australia.

[10] Royal Life Saving Society – Australia , “National Swimming and Water Safety Framework,” 2020, Royal Life Saving Society – Australia.

[11] Royal Life Saving Society – Australia , “Work Needed to Catch up on Missed Swimming Lessons,” 2022, Royal Life Saving Society – Australia, https://www.royallifesaving.com.au/about/news‐and‐updates/news/2022/oct/work‐needed‐to‐catch‐up‐on‐missed‐swimming‐lessons.

[12] S. M. Willcox‐Pidgeon , A. E. Peden , and J. Scarr , “Exploring Children's Participation in Commercial Swimming Lessons Through the Social Determinants of Health,” Health Promotion Journal of Australia 32, no. 2 (2021): 172–181. 32187399 10.1002/hpja.335

[13] P. Larsen , S. Pidgeon , and J. Scarr , Children's Swimming & Water Safety Skills: Teacher and Parent Perceptions (Royal Life Saving Society – Australia, 2025).

[14] NSW Government Office of Sport , “First Lap,” 2023, https://www.sport.nsw.gov.au/firstlap.

[15] R. Macniven , B. Angell , N. Srinivasan , K. Awati , J. Chatman , and A. E. Peden , “Evaluation of the First Lap Learn to Swim Voucher Programme: Protocol,” Injury Prevention 29, no. 2 (2023): 188–194. 36344270 10.1136/ip-2022-044711

[16] R. Macniven , E. Mead , B. Angell , and A. E. Peden , “Population Reach and Redemption of Swimming Lesson Vouchers for Pre‐School‐Aged Children in New South Wales, Australia,” Public Health 248 (2025): 105958. 40967088 10.1016/j.puhe.2025.105958

[17] Python Software Foundation , “Python [Open‐Source Software],” 2023, Version 3.12.0, Python Software Foundation, https://www.python.org/.

[18] S. Seabold and J. Perktold , “Statsmodels: Econometric and Statistical Modeling With Python,” 2010, Proceedings of the 9th Python in Science Conference, https://conference.scipy.org/proceedings/scipy2010/pdfs/seabold.pdf.

[19] J. Fereday and E. Muir‐Cochrane , “Demonstrating Rigor Using Thematic Analysis: A Hybrid Approach of Inductive and Deductive Coding and Theme Development,” International Journal of Qualitative Methods 5, no. 1 (2006): 80–92.

[20] J. Wheeldon and J. Faubert , “Framing Experience: Concept Maps, Mind Maps, and Data Collection in Qualitative Research,” International Journal of Qualitative Methods 8, no. 3 (2009): 68–83.

[21] S. Audrey , B. W. Wheeler , J. Mills , and Y. Ben‐Shlomo , “Health Promotion and the Social Gradient: The Free Swimming Initiative for Children and Young People in Bristol,” Public Health 126, no. 11 (2012): 976–981. 22902210 10.1016/j.puhe.2012.07.008

[22] N. Bolton and S. Martin , “The Policy and Politics of Free Swimming,” International Journal of Sport Policy and Politics 5, no. 3 (2013): 445–463.

[23] S. Bullough , L. E. Davies., and D. Barrett , “The Impact of a Community Free Swimming Programme for Young People (Under 19) in England,” Sport Management Review 18, no. 1 (2015): 32–44.

[24] Royal Life Saving Society – Australia , “National Aquatic Industry Workforce Report 2023,” 2023, Royal Life Saving Society – Australia.

[25] NSW Government Office of Sport , “Take the Plunge and Become a Swim Instructor,” 2022, NSW Government, https://www.sport.nsw.gov.au/media‐releases/take‐plunge‐and‐become‐a‐swim‐instructor.

[26] B. C. Foley , N. Turner , K. B. Owen , D. Cushway , J. Nguyen , and L. J. Reece , ““It Goes Hand in Hand With us Trying to Get More Kids to Play” Stakeholder Experiences in a Sport and Active Recreation Voucher Program,” International Journal of Environmental Research and Public Health 20, no. 5 (2023): 4081. 36901093 10.3390/ijerph20054081 PMC10001936

[27] V. Ananthapavan , A. E. Peden , B. Angell , and R. Macniven , “Barriers to Preschool Aged Children's Participation in Swimming Lessons in New South Wales, Australia,” Health Promotion Journal of Australia 35, no. 3 (2024): 770–783. 37807369 10.1002/hpja.811

[28] E. Mead , A. E. Peden , B. Angell , and R. Macniven , “Challenges to Young Children's Swimming Lesson Participation in New South Wales, Australia,” Injury Prevention 31 (2025): 518–526. 38991716 10.1136/ip-2024-045285

[29] NSW Government Office of Sport , “Learn to Swim Program,” 2025, https://www.sport.nsw.gov.au/learn‐to‐swim‐program.

[30] C. E. Simpson , R. Virgara , R. G. Curtis , et al., “Setting the Game Plan: An International Delphi Study on Evaluating a Population‐Wide Youth Sports Financial Incentive,” BMC Public Health 24, no. 1 (2024): 3295. 39605042 10.1186/s12889-024-20830-0 PMC11600762

[31] L. Miller , “Analysis of Unintentional Drowning in Australia 2002‐2022: Progress, Challenges, and Data to Inform Prevention,” 2023, https://www.royallifesaving.com.au/__data/assets/pdf_file/0016/77200/Royal‐Life‐Saving‐Analysis‐of‐Unintentional‐drowning‐in‐Australia‐2002‐2022_v2_SPG.pdf. 10.1016/j.anzjph.2025.100258 40695639

[32] C. K. Hanum , A. E. Peden , E. Mead , B. Angell , and R. Macniven , “Changes in Parent/Carer Water Safety Awareness During a Swimming Lesson Voucher Programme Implementation in New South Wales, Australia,” Injury Prevention (2025), 10.1136/ip-2025-045701. 40707241

